# Book Reviews

**Published:** 1892-10

**Authors:** 


### *BOOK REVIEWS.*

USES OF WATER IN MODERN MEDICINE. Two volumes. By Simon Baruch, M. D., New York.

CEREBRAL MENINGITIS. By Martin W. Barr, M. D., Elwyn, Pennsylvania.

CONTRIBUTIONS OF PHYSICIANS TO ENGLISH AND AMERICAN LITERATURE. By Robert C. Kenner, M. D., Louisville, Ky.

The above modest works belong to the convenient and valuable series of the "Physicians' Leisure Library," which is issued monthly by Geo. S. Davis, of Detroit.

In the first, Dr. Baruch attempts to define the true function and place of water in the treatment of disease. The author believes water to be a potent remedial agent, but emphasizes the important distinction between *hydrotherapy* and *hydropathy*. In different chapters he gives its mode of action, the technique of its application, the preparation of different baths, its specific application in certain diseases, etc.

The second is a monograph on the varieties of cerebral meningitis. The classification and description of the several forms are somewhat different from those usually found in the books, and appear to be both accurate and thorough.

The third is a highly interesting "little volume, giving an account of the activity of physicians in the field of general literature." The writer's selection of authors is very good, although several well-known names have been omitted. Short sketches of their lives are given, with short selections from their works.

---

A Text-Book of the Principles and Practice of Medicine. For the use of Students and Practitioners. By Henry M. Lyman, M. D., Professor of the Principles and Practice of Medicine, in Rush Medical College, Chicago. In one octavo volume of 926 pp., with 170 illus. Philadelphia, Lea Brothers & Co., 1892.

Dr. Lyman's work on Practice is suited more to the needs of students than practitioners. Brevity has been aimed at in its preparation, and the subject matter is made very compact. Still all subjects usually treated in text-books on Practice are here discussed clearly and concisely. While the author states that matters of a controversial nature have been omitted, he confidently classes measles and chicken-pox among the diseases caused by vegetable parasites. In this and other places Dr. Lyman seems a little anticipatory. The arrangement of topics is somewhat original. "Having acquired a knowledge of the diseases that involve the external surface of the body, of which so many are of a parasitic nature, it seems best to begin the study of internal medicine by continuing the investigation of parasitic diseases." We fail to see where anything is gained by this arrangement. Students will find this a very useful work from the fact that it is essentially a work of to-day. The author has been thoroughly alive to the progress that has been made along different lines of medical knowledge, and has taken pains to give full credit for this when it was indicated.

DISEASES OF THE STOMACH. By Dr. C. A. Ewald, Extraordinary Professor of Medicine at the University of Berlin ; Director of the Augusta Hospital, etc. Authorized Translation from the Second German Edition. By Dr. Morris Manges, New York City. D. Appleton & Co., New York.

The present volume is the second part of the author's treatise on the physiology and pathology of digestion in their practical relations. The first volume appeared three or four years ago, and the favor with which it was received led to its translation in several languages. This volume will probably be as successful as the former. All the diseased conditions of the stomach are exhaustively discussed; the chapters on cancer, ulcer, and the neuroses of the stomach being, perhaps, the best portions of the work. For ulcer of the stomach the author is partial to the rest treatment, and claims that this gives the most satisfactory results. This is a most thorough and practical treatise, and a valuable contribution to the literature of stomach diseases. It is well provided with plates and illustrations. The author has made several important additions to his previous German editions especially for this translation. In spite of the great amount of investigation that has been recently conducted in the diseases of digestion, the author seems to have given full credit for the latest results.

---

PRACTICAL SURGERY; ITS THEORY AND PRACTICE. By William J. Walsham, F. R. C. S., Assistant Surgeon to St. Bartholomew's Hospital; Surgeon to the Metropolitan Free Hospital, London, etc. P. Blakiston, Son & Co., Philadelphia.

This is the third edition of this well known work on practical surgery, and it may well be recommended to the student and practitioner as a condensed and useful treatise.

Chapters on diseases of the eye and ear have been added in this edition. Special prominence is given to the pathology of inflammation in which the author adopts the theory of Virchow, believing it more rational than that of Cohnheim.

The sections on Pathology, Bacteriology and Tubercle have been carefully prepared and extended over former editions. The author attempts to give the science of surgery in as short and condensed a form as possible, avoiding detail and obscure diseases. Its greatest value will be appreciated by students, to whom it seems specially adapted. B. W. B.

---

THE PHYSICIAN HIMSELF. By D. W. Cathell, M. D. Tenth Edition. F. A. Davis Publishing Co., Philadelphia.

This popular work, which has reached its tenth edition, is too well known to require extended notice. It should find a place in every physician's library, and the younger members of the profession will find it to abound in wholesome advice and suggestions which may stand them in good stead on many occasions. The reception which has been accorded Dr. Cathell's book is of greater worth than any words of the reviewer.

---

STUDENT'S HAND-BOOK OF SURGICAL OPERATIONS. By Frederick Treves, F. R. C. S., Surgeon to and Lecturer on Anatomy at the London Hospital, Examiner in Surgery at the University of Cambridge. Lea Brothers & Co., Philadelphia.

This book is essentially a student's volume, and as such will be very useful. It is an abridgment of the author's large "Manual of Operative Surgery." General principles are not discussed, nor critical comparisons made between the value of different methods, but the most essential and commonly performed operations are clearly and concisely described. The omission of anatomical details, descriptions of instruments, directions for after-treatment, etc., made it possible for the author to present nearly the whole subject of operative surgery within the compass of this convenient volume. "Students preparing for final examinations" (for which the work is intended) will find it of much service.

